# Intentions to undergo primary screening with colonoscopy under the National Cancer Screening Program in Korea

**DOI:** 10.1371/journal.pone.0247252

**Published:** 2021-02-24

**Authors:** Kyeongmin Lee, Haejoo Seo, Sunho Choe, Seung-Yong Jeong, Ji Won Park, Mina Suh, Aesun Shin, Kui Son Choi

**Affiliations:** 1 Graduate School of Cancer Science and Policy, National Cancer Center, Goyang-si, Republic of Korea; 2 Department of Preventive Medicine, Seoul National University College of Medicine, Seoul, Republic of Korea; 3 Department of Surgery, Seoul National University College of Medicine, Seoul, Republic of Korea; 4 Cancer Research Institute, Seoul National University, Seoul, Republic of Korea; 5 National Cancer Control Institute, National Cancer Center, Goyang-si, Republic of Korea; Faculty of Health Sciences - Universidade da Beira Interior, PORTUGAL

## Abstract

**Background:**

We sought to investigate intentions to undergo primary screening with colonoscopy in an attempt to predict future colorectal cancer screening behaviors and the feasibility of implementing colonoscopy as the primary screening modality for colorectal cancer in the National Cancer Screening Program (NCSP) of Korea.

**Methods:**

Data were obtained from a nationwide online survey conducted in 2018. The survey included a total of 800 eligible adults aged over 45 years. Study measures included the history of screening colonoscopy within the past 10 years and intentions to undergo primary screening with colonoscopy under the NCSP based on the five constructs of the Health Belief Model. Logistic regression analysis was conducted to examine factors associated with intentions to undergo primary screening with colonoscopy.

**Results:**

Approximately 77% of the participants expressed strong willingness to undergo primary screening with colonoscopy under the NCSP. Higher perceived severity and perceived benefits were significantly associated with stronger intentions to undergo screening with colonoscopy (adjusted odds ratio [aOR], 1.53; 95% confidence interval [CI], 1.10–2.14 and aOR, 2.74; 95% CI, 1.76–4.28, respectively). Greater perceived barriers (aOR, 0.65; 95% CI, 0.45–0.93) were significantly associated with weaker intentions. Cues to action elicited the strongest screening intentions (aOR, 8.28; 95% CI, 5.23–13.12).

**Conclusion:**

The current study findings highlight the need for increasing awareness of the severity of CRC and the benefits of colonoscopy screening. Family-orientated recommendation strategies and reducing complications may boost an individual’s intentions to undergo colonoscopy.

## Background

According to GLOBOCAN 2018, colorectal cancer (CRC) ranks the third most commonly diagnosed malignancy and the second leading cause of cancer death worldwide [1]. In Korea, CRC was the third most frequently occurring cancer among both men and women in 2016 (age-standardized rates of 41.6 per 100,000 and 23.3 per 100,000, respectively) [2]. As most CRCs develop through prolonged transformation of adenomas into carcinomas, early detection is one of the most effective measures through which to reduce CRC burden.

In Korea, the National Cancer Screening Program (NCSP) was launched initially for gastric, breast, and cervical cancers without cost to medical aid beneficiaries in 1999. Since 2004, the NCSP has provided annual fecal occult blood test (FOBT) as the primary CRC screening modality for adults aged 50 years or older. People with positive results from the FOBT are further referred to undergo follow-up colonoscopy or a double-contrast barium enema test [3]. In addition to the NCSP, FOBT and colonoscopy are also conducted in outpatient or private health-assessment centers as options in opportunistic screening.

High uptake among the target population in an organized cancer screening program is necessary to reduce cancer mortality, and the lack of participation in a screening program can jeopardize its effectiveness [4]. According to the NCSP database in Korea from 2002 to 2012, the participation rate for CRC screening was the lowest among all cancers [3]. Several potential reasons may account for this low rate: First is the screening process for FOBT. To undergo FOBT, invited individuals are required to visit a CRC screening unit once to collect the kit and again to return it, which may inconvenience the participants [3]. Second may be an increasing demand for opportunistic screening colonoscopies among eligible individuals. Indeed, according to the Korean National Cancer Screening Survey (KNCSS), screening rates for opportunistic colonoscopy have increased up to 45.5%, whereas those for FOBT in either organized or opportunistic screening have remained between 25% and 30%, as of 2018 [5].

Apart from its high accuracy, colonoscopy is considered the gold standard of CRC screening because it can detect and remove colorectal polyps during the same procedure through direct visualization of the entire colon [6]. Due to this advantage, colonoscopy has been adopted as the primary CRC screening modality in several countries, including Austria, Germany, Poland, Switzerland, and the United States [7]. Accordingly, in 2019, the Korean Colonoscopy Screening Project began to examine the feasibility of implementing colonoscopy as the primary CRC screening modality under the NCSP. However, its feasibility has proven controversial due to potential risks of perforation and hemorrhage [8]. In addition, bowel preparation and a complicated examination process have been reported as sources of unsatisfactory experiences with colonoscopy [9]. Nevertheless, the cost of colonoscopy in Korea is very low, compared to other countries, and the number of hospitals and clinics that offer quality colonoscopy through opportunistic examinations is increasing [10]. Thus, in terms of colonoscopy costs and accessibility, Korea is in a much better situation to offer colonoscopy as the primary CRC screening modality than other countries [11].

The Health Belief Model (HBM) was developed in the early 1950s in an attempt to better understand the widespread failure of people to accept disease preventives or screening tests for the early detection of asymptomatic disease [12]. In Korea, although numerous studies have identified various factors associated with CRC screening [13–16], only a few studies have used the HBM to identify associations between an individual’s health beliefs and CRC screening with FOBT [17, 18], and only one study has investigated associations between health beliefs and screening with colonoscopy [19].

Thus, we sought to investigate intentions to undergo primary screening with colonoscopy in order to estimate future CRC screening behaviors and the feasibility of implementing colonoscopy as the primary screening option for CRC. Additionally, as colonoscopy has been widely administered in Korea as either a confirmatory test after positive FOBT results or for opportunistic screening, we also examined associations between an individual’s health beliefs, including perceived susceptibility, perceived severity, perceived benefits, perceived barriers, and cues to action, and screening intentions according to prior experiences with colonoscopy.

## Methods

### 1. Study design and subjects

A nationwide online survey was conducted to examine intentions to undergo primary screening with colonoscopy among eligible individuals in 2018. Although many guidelines recommend an eligible age for CRC screening of 50 years and older, the Korea National Cancer Screening Guidelines recommend CRC screening with annual FOBT or selective use of colonoscopy for adults aged 45 to 80 years due to an increased risk of CRC in Koreans after age 45 [20]. Thus, in consideration thereof, the survey was sent to adults targeted for screening aged 45 years and older via a link to an online questionnaire, and data were collected by a professional research agency from July 20 to August 6, 2018. The time required to complete the survey was less than 10 minutes, and the completeness of the questionnaires was checked by a researcher. The sample size was estimated using the G*power 3.1.9.7 program. Based on a significance level (α) of 0.05, a statistical power (1-β) of 0.95, an effect size medium (0.15), and the number of predictors set to 16, the appropriate sample size was calculated to be 204 [21]. Finally, a nationally representative random sample, including 800 participants aged 45 to 80 years, was randomly selected through a stratified multistage sampling according to demographic characteristics, including geographical area, age, and sex.

### 2. Measures

The survey questionnaire was designed to collect information on socio-demographic characteristics, previous history of colonoscopy screening, health beliefs, and intentions to undergo primary screening with colonoscopy under the NCSP. Previous studies that investigated psychological factors associated with CRC screening with FOBT can be found elsewhere [17, 18]. As we mainly aimed to investigate factors influencing screening with colonoscopy, the study participants’ screening behaviors associated with screening with FOBT were not measured.

The HBM was originally developed for studying and promoting the uptake of health services [22]. In this study, the HBM scales were revised from Jung’s Korean version of the HBM scale, which was revised and refined from prior HBM subscales, including those proposed by Lee et al. and Rawls et al [19, 23, 24]. The HBM comprised a total of 33 items in the areas of five major constructs: perceived susceptibility, perceived severity, perceived benefits, perceived barriers, and cues to action. Regarding perceived susceptibility, four items were used to measure an individual’s perceived risk or chances of contracting CRC. Perceived severity, which is a subjective measure of seriousness of contracting CRC and its negative consequences, was measured by seven items. Regarding perceived benefits, five items were used to gauge subjective beliefs about the usefulness of colonoscopy. Regarding perceived barrier, eleven items were used to identify barriers to undergoing colonoscopy. Finally, regarding cues to action, there were six items for measuring both internal and external triggers for undergoing colonoscopy. The reliability indices of the research instrument (Cronbach’s alpha coefficients) were calculated for perceived susceptibility (0.88), perceived severity (0.86), perceived benefits (0.87), perceived barriers (0.83), and cues to action (0.80) (S2 Table).

Fifteen questions were designed to collect information on each subject’s age, sex, residential area, education years, marital status, occupation, monthly household income, private cancer insurance status, health consciousness, physical activity, smoking status, chronic disease, history of cancer diagnosis, family history of cancer, and CRC screening recommendation. Recent history of colonoscopy screening was assessed by asking “Have you undergone screening colonoscopy during last 10 years?” Individuals who underwent screening were defined as the up-to-date group, while those who did not were defined as the not up-to-date group.

Prior to examining screening intentions, each participant was given general information on colonoscopy, describing what colonoscopy is, what the test involves, and the benefits and harms of colonoscopy. Then, the participants were asked, “If colonoscopy is made available as the primary CRC screening modality under the NCSP, would you like to undergo colonoscopy?” Subjective health beliefs on colonoscopy and intentions to undergo primary screening with colonoscopy were assessed using a five-point Likert scale (strongly disagree = 1, disagree = 2, neutral = 3, agree = 4, and strongly agree = 5).

### 3. Statistical analysis

First, descriptive statistics were applied to examine socio-demographic characteristics, mean and standard deviations of each construct of the HBM, and screening intentions. The socio-demographic characteristics were compared according to recent history of screening colonoscopy using the chi-square test for categorical variables and ANOVA for continuous variables. Second, principal axis factor analysis was conducted to classify each item in the HBM (S1 Table). Hair et al. suggested an acceptable factor loading value to be more than 0.5 and ideally 0.7 or higher [25]. Thus, questions with a factor loading <0.70 were excluded from the analysis. Third, associations between an individual’s health beliefs and previous history of colonoscopy were tested using independent samples t-test. Bonferroni correction was used for multiple comparisons, and the significance level was set at *P* value <0.002.

Finally, logistic regression analysis was conducted to examine factors associated with intentions to undergo primary screening with colonoscopy under the NCSP. In the analysis, screening intentions were categorized as binary outcomes. Those who answered either strongly disagree, disagree, or neutral were categorized as having “weak intentions,” while those who answered either agree or strongly agree were categorized as having “strong intentions.” Variance of inflation factor (ranging from 0.4 to 1.5) indicated no multicollinearity problems. C-statistic (0.87) and the Hosmer-Lemeshow goodness-of-fit test indicated that this model fit the data well (*P* value = 0.29). A result was considered statistically significant at *P* value <0.05.

Further, sub-group analysis was conducted to highlight associations between screening intentions and a previous history of colonoscopy. All statistical analyses were conducted using STATA software (version. 13.1, College Station, Texas 77845 USA).

### 4. Ethical statement

This study was approved by Institutional Review Board of Seoul National University Hospital in July 2018 (C-1806-094-952). Upon IRB approval, a link to the online survey was sent to the study participants. Among those who received a link, they were asked to read the instructions and were given an opportunity to accept or decline the survey. Only those who agreed to provide informed consent by clicking “agree” on the instruction page were able to participate in the survey.

## Results

The lifetime screening rate and the screening rate of colonoscopy within the last 10 years among the study participants are presented in S1 and S2 Figs, respectively. In general, we noted that the colonoscopy screening rate was significantly higher in men aged between 45 and 65 years than in women of the same ages. In women, the screening rate gradually increased with increasing age, and screening rates were the highest among women older than 65 years, higher than those in men of the same.

The mean scores for the questions on the five HBM constructs were compared according to up-to-date status for colonoscopy (Table 1). In the up-to-date group, mean scores for perceived susceptibility, perceived benefits, and cues to action were significantly higher, while scores for perceived barriers were significantly lower than those in the not-up-to date group (all *P* values <0.001). Regardless of up-to-date status, mean scores for chances of developing CRC in one’s lifetime (perceived susceptibility), complete change of life upon getting CRC (perceived severity), usefulness of colonoscopy in early detection of CRC (perceived benefits), pain and fear of complications from colonoscopy (perceived barrier), and getting recommendations to undergo colonoscopy from a family member or friends (cues to action) were highest.

**Table 1:** Differences in health beliefs according to recent colonoscopy screening.

	No colonoscopy screening within 10 years	Colonoscopy screening within 10 years	*P* value^a)^
	Mean±SD	Mean±SD	
**Perceived susceptibility**	**2.96±0.75**	**3.15±0.75**	<0.001*
Chance of developing CRC in one’s lifetime	3.39±0.88	3.55±0.79	0.006
Chance of developing CRC within 10 years	2.91±0.87	3.07±0.86	0.010
Having many risk factors for CRC	2.87±0.88	3.08±0.91	0.002
Probability of developing CRC compared to others	2.68±0.91	2.90±0.94	0.001*
**Perceived severity**	**3.71**±**0.78**	**3.77**±**0.76**	**0.293**
CRC causes long lasting problems.	3.59±0.96	3.71±0.91	0.073
CRC will negatively affect family and social relationships.	3.70±0.98	3.77±0.94	0.305
CRC will completely change my life.	3.85±0.91	3.89±0.93	0.464
CRC treatment is expensive.	3.71±0.87	3.70±0.84	0.917
**Perceived benefits**	**3.93**±**0.54**	**4.09**±**0.59**	<0.001*
High chance of survival if CRC is found early	3.92±0.68	4.07±0.69	0.002
Colonoscopy helps early detection of CRC.	4.10±0.64	4.27±0.71	0.001*
Treatment for CRC is not difficult if it is found early.	3.84±0.75	3.95±0.72	0.034
Colonoscopy will reduce concerns about CRC.	3.87±0.68	4.10±0.71	<0.001*
Colonoscopy will help reduce CRC deaths.	3.93±0.67	4.05±0.76	0.014
**Perceived barriers**	**3.03±0.67**	**2.47±0.79**	<0.001*
Colonoscopy is expensive.	2.85±0.93	2.42±0.96	<0.001*
Do not know what colonoscopy is	3.02±0.95	2.26±1.03	<0.001*
Colonoscopy is painful.	3.37±0.87	2.64±1.05	<0.001*
Complications from colonoscopy	3.33±0.96	2.77±1.00	<0.001*
Transportation difficulties	2.58±0.94	2.26±0.96	<0.001*
**Cues to action**	**3.35±0.63**	**3.85±0.63**	<0.001*
Recommendation from family or friends	3.44±0.75	3.95±0.71	<0.001*
Recommendation from mass media	3.22±0.77	3.70±0.78	<0.001*
Concerns about health status	3.39±0.78	3.90±0.77	<0.001*

SD, standard deviation.

^a)^Comparison of mean scores on each question of the five HBM constructs according to recent experiences with colonoscopy screening using t-test.

*Significant at *P* value <0.002 (Bonferroni correction was used for multiple comparisons).

**Fig 1:**
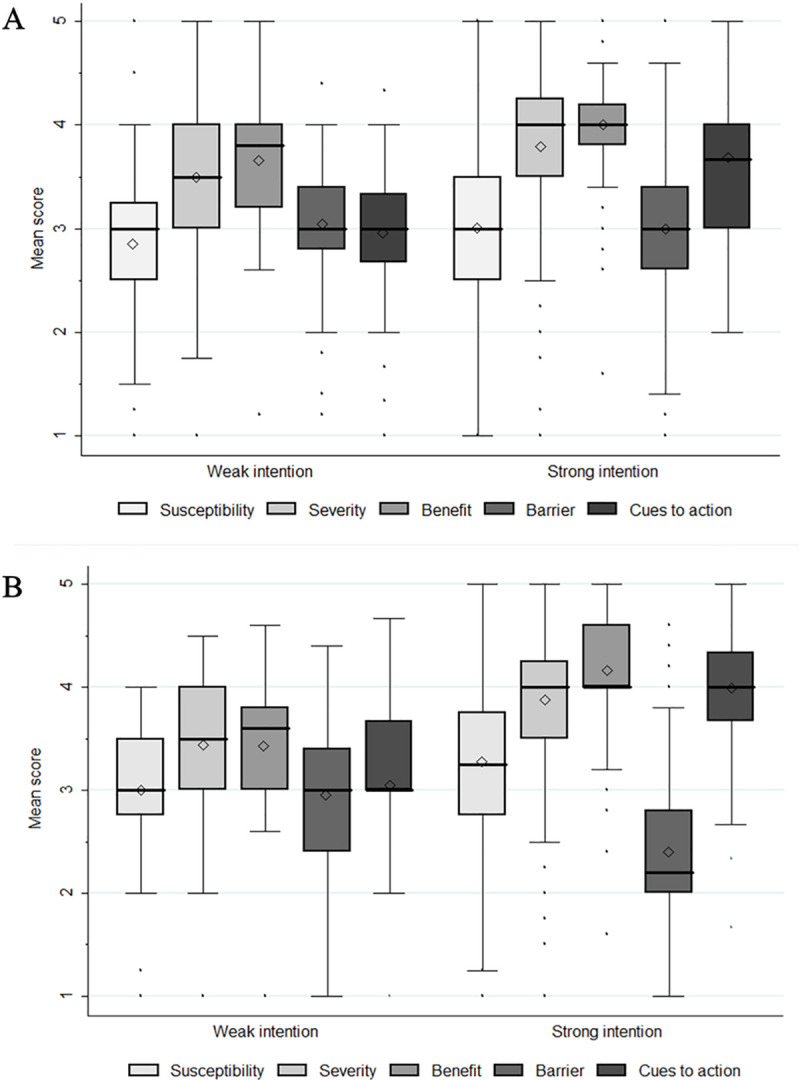
Comparison of mean, median, and interquartile range values for the five health belief constructs according to weak/strong screening intentions stratified by up-to-date status of colonoscopy screening. (A) Comparing health beliefs among the not-up-to date group; (B) comparing health beliefs among the up-to-date group. ◇ indicates mean values.

Fig 1 shows the mean, median, and interquartile range values for the five health belief constructs according to strong/weak intentions to undergo screening stratified by up-to-date status for colonoscopy screening. Among the not up-to-date group, we noted that people with stronger intentions had significantly higher mean and median scores for perceived severity and cues to action, compared to people with weak intentions. Meanwhile, among the up-to-date group, mean and median scores for perceived susceptibility, perceived severity, perceived benefit, and cues to action were significantly higher, while those for perceived barrier were significantly lower for people with stronger intentions than people with weak intentions.

The number of study participants with strong intentions to undergo primary screening colonoscopy under the NCSP and factors associated with strong intentions are presented in Table 2. Among the participants who were not up-to-date with colonoscopy, 68.1% reported having strong intentions, whereas 87.5% of the up-to-date group expressed strong willingness to undergo colonoscopy. Among the not-up-to date group, having private cancer insurance was significantly associated with strong intentions (*P* value = 0.001), while a high monthly household income (*P* value <0.001) was a significantly associated with strong intentions among the up-to-date group.

**Table 2:** Intentions to undergo colonoscopy under the NCSP according to previous history of colonoscopy (n = 800).

Variable	No colonoscopy screening within 10 years (n = 423)			Colonoscopy screening within 10 years (n = 377)		
	Intention, n (%)^a)^		*P* value^b)^	Intention, n (%)^a)^		*P* value^b^
	Strong	Low		Strong	Low	
**Total**	288 (68.1)	135 (31.9)		330 (87.5)	47 (12.5)	
**Age group (years)**			0.300			0.701
45–54	139 (70.6)	58 (29.4)		105 (86.1)	17 (13.9)	
55–64	106 (68.4)	49 (31.6)		137 (87.3)	20 (12.7)	
65–78	43 (60.6)	28 (39.4)		88 (89.5)	10 (10.2)	
**Residential area**			0.570			0.570
Metropolitan	131 (66.2)	67 (33.8)		155 (88.6)	20 (11.4)	
Non-metropolitan	157 (69.8)	68 (30.2)		175 (86.6)	27 (13.4)	
**Sex**			0.326			0.075
Male	132 (70.6)	55 (29.4)		186 (90.3)	20 (9.7)	
Female	156 (66.1)	80 (33.9)		144 (84.2)	27 (15.8)	
**Years of education**			0.334			0.080
6–12 years	95 (65.1)	51 (34.9)		80 (82.5)	17 (17.5)	
More than 13 years	193 (69.7)	84 (30.3)		250 (89.3)	30 (10.7)	
**Monthly household income**			0.137			<0.001
Less than $2,999	83 (61.5)	52 (38.5)		48 (85.7)	19 (28.4)	
$3,000~$4,999	111 (71.2)	45 (28.8)		124 (89.9)	14 (10.1)	
More than $5,000	94 (71.2)	38 (28.8)		158 (91.9)	14 (10.1)	
**Employment status**			0.483			0.138
Unemployed	78 (65.6)	41 (34.5)		61 (82.4)	13 (17.6)	
Employed	210 (69.1)	94 (30.9)		269 (88.8)	34 (11.2)	
**Physical activity**			0.495			0.57
None	52 (62.7)	31 (37.3)		48 (85.7)	8 (14.3)	
Moderate	156 (69.3)	69(30.7)		165 (86.4)	26 (13.6)	
Regular	80 (69.6)	35 (30.4)		117 (90.0)	13 (10.0)	
**Private cancer insurance**			0.001			0.061
No	61 (55.0)	50 (45.0)		60 (81.1)	14 (18.9)	
Yes	227 (72.8)	85 (27.2)		270 (89.1)	33 (10.9)	
**Smoking status**			0.183			0.811
No	211 (66.4)	107 (33.6)		226 (87.3)	33 (12.7)	
Yes	77 (73.3)	28 (26.7)		104 (88.1)	14 (11.9)	
**Chronic Disease**			0.531			0.338
No	183 (67.0)	90 (33.0)		172 (86.0)	28 (14.0)	
Yes	105 (70.0)	45 (30.0)		158 (89.3)	19 (10.7)	

Chronic disease, being diagnosed with any of following diseases: hypertension, diabetes, chronic kidney disease, cerebrovascular disease, and inflammatory bowel disease.

^a)^Row percentage for strong intention, participants either agreed or strongly agreed to undergo primary screening with colonoscopy, while those who answered either strongly disagree, disagree, or neutral were categorized as having weak intentions.

^b)^Comparison of proportions of people with strong and weak intentions using the chi-square test.

Table 3 indicates the factors associated with intentions to undergo colonoscopy. Results from adjusted odds ratios (aOR) showed that the odds of having strong intentions to undergo screening with colonoscopy increased 1.53 fold (95% CI, 1.10–2.14) and 2.74 fold (95% CI, 1.76–4.28), respectively, with one unit increase in perceived severity and perceived benefit and decreased 0.65 fold (95% CI, 0.45–0.93) with one unit increase in perceived barrier. The odds of strong intentions was highest with one unit increase in cues to action (aOR, 8.28; 95% CI, 5.23–13.12).

**Table 3:** Factors associated with intentions to undergo primary colonoscopy under the NCSP (n = 800).

Variables	OR	95% CI	aOR	95% CI
**Age group (years)**				
45–54	1.00	reference	1.00	reference
55–64	1.08	0.75–1.57	0.92	0.57–1.48
65–78	1.06	0.68–1.65	0.80	0.44–1.47
**Residential area**				
Metropolitan	1.00	reference	1.00	reference
Non-metropolitan	1.06	0.76–1.48	1.15	0.76–1.75
**Sex**				
Male	1.00	reference	1.00	reference
Female	0.66	0.47–0.92	0.90	0.56–1.47
**Years of education**				
6–12 years	1.00	reference	1.00	reference
More than 13 years	1.51	1.07–2.14	0.84	0.52–1.35
**Monthly household income**				
Less than $2,999	1.00	reference	1.00	reference
$3,000~$4,999	2.16	1.44–3.24	1.30	0.77–2.21
More than $5,000	2.63	1.73–3.98	1.08	0.61–1.91
**Employment status**				
Unemployed	1.00	reference	1.00	reference
Employed	1.45	1.00–2.10	1.16	0.69–1.94
**Physical activity**				
None	1.00	reference	1.00	reference
Moderate	1.32	0.85–2.04	1.14	0.65–2.00
Regular	1.60	0.98–2.60	1.23	0.65–2.34
**Private cancer insurance**				
No	1.00	reference	1.00	reference
Yes	2.23	1.55–3.20	1.36	0.83–2.22
**Smoking status**				
No	1.00	reference	1.00	reference
Yes	1.38	0.94–2.03	1.00	0.59–1.70
**Chronic Disease**				
No	1.00	reference	1.00	reference
Yes	1.37	0.97–1.93	1.16	0.74–1.82
**Colonoscopy screening within 10 years**				
No	1.00	reference	1.0	reference
Yes	3.29	2.28–4.75	1.53	0.94–2.47
**Perceived susceptibility**^**a)**^	1.54	1.23–1.92	0.84	0.59–1.19
**Perceived severity**^**a)**^	1.90	1.54–2.35	1.53	1.10–2.14
**Perceived benefit**^**a)**^	5.48	3.80–7.91	2.74	1.76–4.28
**Perceived barrier**^**a)**^	0.57	0.46–0.72	0.65	0.45–0.93
**Cues to action**^**a)**^	11.05	7.32–16.70	8.28	5.23–13.12

OR, crude Odds Ratio; aOR, adjusted Odds Ratio; 95% CI, 95% Confidence Interval; Chronic disease, being diagnosed with any of following diseases: hypertension, diabetes, chronic kidney disease, cerebrovascular disease, and inflammatory bowel disease.

^**a)**^OR per one score increase.

The socio-demographic characteristics of the study participants according to recent experiences with colonoscopy screening are listed in S3 Table. Of the 800 participants, 377 (47.1%) responded as having undergone colonoscopy during last 10 years. Those who had undergone colonoscopy were significantly more likely to be older, male, regular exercisers, and current smokers and to have private cancer insurance, a chronic disease, a higher household income, and a higher education, compared to those who had not undergone colonoscopy.

Further sub-group analysis was conducted to highlight associations between screening intentions and previous screening history of colonoscopy (S4 Table). Among participants who were not up-to-date with colonoscopy, the odds of a strong intention significantly increased 1.66 fold (95% CI, 1.11–2.49) with one unit increase in perceived severity. Regardless of previous screening with colonoscopy, higher perceived benefit (aOR, 2.25; 95% CI, 1.31–3.86 and aOR, 5.28; 95% CI, 2.20–12.65, respectively) and higher cues to action (aOR, 8.48; 95% CI, 4.77–15.09 and aOR, 8.00; 95% CI, 3.33–19.22, respectively) were significantly associated with stronger intentions to undergo primary screening with colonoscopy.

## Discussion

In the present study, we described the screening rates of colonoscopy and examined factors associated with intentions to undergo the primary screening with colonoscopy among eligible individuals in the NCSP using the HBM. According to KNCSS data for 2018, the screening rate of colonoscopy gradually increased from 30.1% in 2012 to 45.4%, while the screening rate of FOBT remained between 25% and 30% [5, 26]. Our study results showed that 47.1% of the participants had undergone colonoscopy during the last 10 years and that approximately 77% had strong intentions to undergo primary screening with colonoscopy if it were implemented in the NCSP. This may indicate that preferences for colonoscopy for CRC screening among people eligible for screening in the NCSP have significantly increased. Nevertheless, despite a significant increase in the uptake of colonoscopy, in Korea, screening rates for colonoscopy have remained relatively low in comparison to other countries: according to the 2015 National Health Interview Survey in the US, 60.3% of eligible adults reported having undergone CRC screening using either flexible sigmoidoscopy within the past 5 years or colonoscopy within the past 10 years [27].

The current study demonstrated that screening rates among younger women were significantly lower than those in men (S1 and S2 Figs). Studies examining the use of colonoscopy have generally showed that women are less likely to undergo colonoscopy than men [28–30]: embarrassment and feelings of vulnerability were most frequently reported as reasons for not undergoing colonoscopy, particularly among women [31]. A few studies have found that female patient preferences for a female colonoscopist have increased significantly and that women who preferred female colonoscopists were more likely to be young and single (P<0.0001) and less likely to be screened [32–34]. In another study, significantly more women than men reported fearing a positive diagnosis and expressed concerns for pain and risks with colonoscopy [35]. Therefore, increasing the availability of female endoscopists and implementing strategies to reduce anxiety among women beforehand while enhancing comfort and modesty during the examination could yield higher screening rates.

In agreement with previous studies, we found that those who are compliant with colonoscopy are more likely to be older and to be of higher socioeconomic status, with private health insurance, higher household income, and higher education, compared to those who are not compliant with colonoscopy [13–16]. Low socioeconomic status maybe associated with an increased risk of CRC because these individuals have less access to cancer screening, less knowledge about the benefits of CRC screening, and no private health insurance. Thus, we suspect that providing appropriate recommendation strategies in consideration of an individual’s socioeconomic status and previous history of CRC screening would help facilitate increases in uptake of colonoscopy screening among eligible individuals.

In this study, we compared the proportions of people with strong intentions to undergo primary screening with colonoscopy under the NCSP and factors associated therewith according to screening status within the previous 10 years. We found that the proportion of people with strong intentions was significantly higher among individuals who were up-to-date with colonoscopy (87.5% vs. 68.1%). This may suggest that previous screening with colonoscopy can affect future screening intentions. Moreover, among the people who were not up-to-date with screening, those with strong intentions to undergo primary screening with colonoscopy under the NCSP were more likely to possess private cancer insurance than those with weaker intentions. One possible explanation for this is that in opportunistic screening, all costs to undergo colonoscopy are to be paid entirely by its users without governmental subsidies [13]. Thus, possessing private cancer insurance may boost intentions among individuals with low household income, as it can reduce the financial burden of undergoing screening. Indeed, people with strong intentions among the up-to-date group were more likely to have a higher monthly household income, potentially because they faced less financial constraint to undergo colonoscopy.

Finally, we investigated factors associated with screening intentions using the HBM. The results showed that higher perceived severity and perceived benefits were significantly associated with stronger intentions to undergo colonoscopy. A few studies have also reported that perceived severity and perceived benefits were significant facilitators of CRC screening [36, 37]. Thus, increasing awareness of the negative impacts of CRC and knowledge and awareness of the effectiveness of colonoscopy in the early detection of CRC ought to boost future intentions among people eligible for screening. Additionally, we found that the odds of strong intentions increased most significantly with higher cues to action, indicating that cues to action had the greatest impact on intentions to undergo screening. Our study results showed that getting recommendations for CRC screening from a family member or friend had the greatest impact on screening intentions. According to Bae et al. (2008), the likelihood of screening significantly increased 4.93 times when cancer screening was frequently recommended by family members [38]. Therefore, family-oriented recommendation strategies for CRC screening will be necessary. Although we considered healthcare provider recommendations in the cues to action subscale, which was previously found to be an important determinant of screening intention [39], it was excluded from the final analysis due to low factor loading in factor analysis.

Meanwhile, a higher perceived barrier was significantly associated with weaker screening intentions. Our study results showed that a fear of complications was the most burdensome part of undergoing colonoscopy, similar to other studies [40, 41]. A population-based study in Canada found that the risk of complications, such as perforation and bleeding, increased threefold with colonoscopists who performed fewer than 300 colonoscopies per year [42]. For this reason, setting a threshold for lifetime experience and a minimum annual number of screening colonoscopies is recommended to ensure the quality of screening colonoscopy in national screening programs [43]. However, in Korea, these standards are set lower than those of other countries [42, 44, 45]. Therefore, increasing quality standards for screening colonoscopists will not only minimize complications from the procedures, but also improve completeness of screening colonoscopy.

As one of our limitations, we used an online survey to examine health beliefs in colonoscopy. Although the study participants were selected through stratified random sampling, only subjects with good computer literacy were able to participate in the survey, which may pose selection bias. Second, the information given to the participants did not fully reflect the characteristics of primary screening colonoscopy under the NCSP (e.g., cost). If colonoscopy becomes available as the primary CRC screening modality under the NCSP, we expect that screening intentions would further increase, especially among people who perceived the cost of colonoscopy to be a significant barrier. Lastly, although a substantial body of research supports the validity of screening intentions to predict future screening behavior, further studies will be necessary to assess actual screening behaviors based on screening intentions. Despite these limitations, to our knowledge, this is the first population-based study to investigate intentions to undergo the primary screening with colonoscopy under the NCSP among eligible individuals using the HBM.

## Conclusions

In summary, we found that most of the study participants had strong willingness to undergo colonoscopy for primary CRC screening under the NCSP. In the current study, among the five constructs of the HBM, perceived severity, perceived benefits, perceived barriers, and cues to action were significantly associated with strong intentions to undergo colonoscopy. Our findings suggest a need for increasing awareness about the negative impacts of CRC and the benefits of colonoscopy. Family-oriented recommendation strategies and reducing complications from colonoscopy may boost an individual’s screening intentions. We also found that psychological factors affecting an individual’s intentions differed according to how up-to-date they were with colonoscopy screening (screening within 10 years). Thus, we suggest recommendation strategies ought to be tailored according to previous history of colonoscopy.

## Supporting information

S1 Fig Lifetime screening rate of colonoscopy. Lifetime screening rate of colonoscopy was defined as having ever undergone screening colonoscopy during one’s lifetime.
(DOCX)

S2 Fig Screening rate of colonoscopy with recommendation. Screening rate of colonoscopy with recommendation was defined as having undergone screening colonoscopy within 10 years in accordance with national cancer recommendations.
(DOCX)

S1 File Questionnaire (English). (DOCX)

S2 File Questionnaire (original). (DOCX)

S1 Table Results of principal axis factor analysis. (DOCX)

S2 Table Internal consistency reliability. SD, standard deviations.
(DOCX)

S3 Table Socio-demographic characteristics of the participants. (DOCX)

S4 Table Intentions to undergo colonoscopy under the NCSP according to screening status. aOR, adjusted odds ratio; 95% CI, 95% confidence interval; Chronic disease, being diagnosed with any of the following diseases: hypertension, diabetes, chronic kidney disease, cerebrovascular disease, and inflammatory bowel disease. * OR per one score increase.
(DOCX)
