# Supplementary figures and images for: Intentions to undergo primary screening with colonoscopy under the National Cancer Screening Program in Korea
Source: PLoS One. 2021 Feb 24;16(2):e0247252. doi: 10.1371/journal.pone.0247252 (PMC7904222; doi:10.1371/journal.pone.0247252)

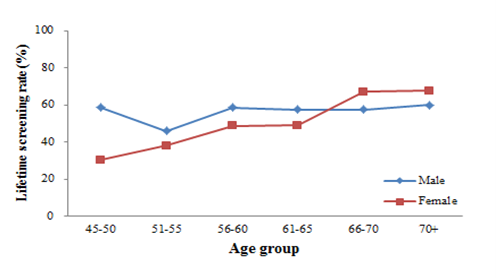


**S1 Fig.**

Supplement: S1 Fig — Lifetime screening rate of colonoscopy was defined as having ever undergone screening colonoscopy during one’s lifetime. (DOCX) [file pone.0247252.s001.docx]

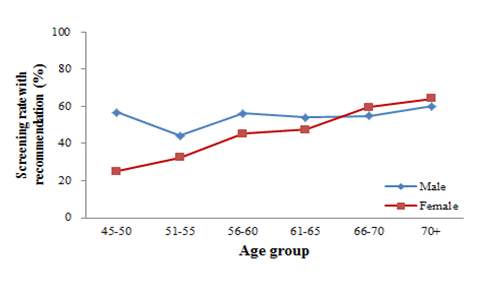


S2 Fig.

Supplement: S2 Fig — Screening rate of colonoscopy with recommendation was defined as having undergone screening colonoscopy within 10 years in accordance with national cancer recommendations. (DOCX) [file pone.0247252.s002.docx]
