# Supplementary material for: Intentions to undergo primary screening with colonoscopy under the National Cancer Screening Program in Korea
Source: PLoS One. 2021 Feb 24;16(2):e0247252. doi: 10.1371/journal.pone.0247252 (PMC7904222; doi:10.1371/journal.pone.0247252)
Supplement: S1 File — (DOCX) [file pone.0247252.s003.docx]

**S1 File.**

| **Section1. Health beliefs on colorectal cancer (CRC) and colonoscopy screening** |
| --- |

**A. Colorectal cancer**

Q1. The questions below are about **perceived susceptibility** of colorectal cancer. Please **tick √ the most appropriate response.**

| **Questions** | | **Strongly disagree** | **Disagree** | **Neutral** | **Agree** | **Strongly agree** |
| --- | --- | --- | --- | --- | --- | --- |
| 1) | It is likely that I will get CRC | ① | ② | ③ | ④ | ⑤ |
| 2) | It is likely that I will get CRC within the next 10 year | ① | ② | ③ | ④ | ⑤ |
| 3) | I have many risk factors for CRC | ① | ② | ③ | ④ | ⑤ |
| 4) | I have higher probability of developing CRC compared to others | ① | ② | ③ | ④ | ⑤ |

Q2. The questions below are about **perceived severity** of colorectal cancer. Please **tick √ the most appropriate response.**

| **Questions** | | **Strongly disagree** | **Disagree** | **Neutral** | **Agree** | **Strongly agree** |
| --- | --- | --- | --- | --- | --- | --- |
| 1) | The thought of getting CRC scares me | ① | ② | ③ | ④ | ⑤ |
| 2) | Problems I would experience with CRC would last a long time | ① | ② | ③ | ④ | ⑤ |
| 3) | CRC will negatively affect family and social relationships | ① | ② | ③ | ④ | ⑤ |
| 4) | If I had CRC, my whole life would change | ① | ② | ③ | ④ | ⑤ |
| 5) | If I had CRC, I would not live longer than 5 years | ① | ② | ③ | ④ | ⑤ |
| 6) | CRC is deadly even if it is found early | ① | ② | ③ | ④ | ⑤ |
| 7) | CRC treatment is expensive | ① | ② | ③ | ④ | ⑤ |

**B. Colonoscopy**

| **What is colonoscopy?** |  |  |
| --- | --- | --- |
|  |  |  |
| Colonoscopy is the endoscopic examination of the large bowel with a small camera on a flexible tube passed through the anus. Colonoscopy is a highly accurate test and if suspicious lesions are found during the procedure, biopsy and endoscopic removal of small polyps can be performed at the same time. However, it is necessary to have bowel preparations to empty your colon before the procedure, which may cause discomfort. You may feel pain or discomfort because the endoscopic tube is inserted through the rectum and advanced to the other end of the large bowel; however, the pain can be reduced through the sedation. Complications from colonoscopy such as bowel perforations (3.8 per 100,000), bleedings from biopsy, abdominal pain as well as acute cardiovascular diseases (e.g. myocardial infarction) among high risk groups including elderly adults and patients with hypertension may occur. | | |

Q3. The questions below are about **perceived benefit** of colonoscopy. Please **tick √ the most appropriate response.**

| **Questions** | | **Strongly disagree** | **Disagree** | **Neutral** | **Agree** | **Strongly agree** |
| --- | --- | --- | --- | --- | --- | --- |
| 1) | Finding CRC early will increase a chance of survival | ① | ② | ③ | ④ | ⑤ |
| 2) | Colonoscopy will help me to find CRC early | ① | ② | ③ | ④ | ⑤ |
| 3) | Treatment for CRC is not difficult if it is found early | ① | ② | ③ | ④ | ⑤ |
| 4) | Colonoscopy will reduce concerns about CRC | ① | ② | ③ | ④ | ⑤ |
| 5) | Colonoscopy will decrease chance of dying of CRC | ① | ② | ③ | ④ | ⑤ |

Q4. The questions below are about **perceived barriers** of colorectal cancer. Please **tick √ the most appropriate response.**

| **Questions** | | **Strongly disagree** | **Disagree** | **Neutral** | **Agree** | **Strongly agree** |
| --- | --- | --- | --- | --- | --- | --- |
| 1) | I am afraid I will find out there is something wrong with me | ① | ② | ③ | ④ | ⑤ |
| 2) | Colonoscopy is embarrassing | ① | ② | ③ | ④ | ⑤ |
| 3) | I do not have enough time to have colonoscopy | ① | ② | ③ | ④ | ⑤ |
| 4) | I can’t afford colonoscopy | ① | ② | ③ | ④ | ⑤ |
| 5) | I do not need colonoscopy because nothing is wrong with me | ① | ② | ③ | ④ | ⑤ |
| 6) | I'm afraid to have colonoscopy because I do not understand what will be done in the test | ① | ② | ③ | ④ | ⑤ |
| 7) | Colonoscopy is painful | ① | ② | ③ | ④ | ⑤ |
| 8) | Preparing for colonoscopy (bowel preparation/diet restriction) is too difficult | ① | ② | ③ | ④ | ⑤ |
| 9) | I'm afraid to have colonoscopy because of possible complications such as intestinal bleeding or intestinal damage. | ① | ② | ③ | ④ | ⑤ |
| 10) | It is difficult to get transportation to have colonoscopy | ① | ② | ③ | ④ | ⑤ |
| 11) | I do not trust colonoscopy | ① | ② | ③ | ④ | ⑤ |

**C. Colorectal cancer screening**

Q5. The questions below are about **cues to action** for colorectal cancer screening. Please **tick √ the most appropriate response.**

| **Questions** | | **Strongly disagree** | **Disagree** | **Neutral** | **Agree** | **Strongly agree** |
| --- | --- | --- | --- | --- | --- | --- |
| 1) | I would have colonoscopy if the doctor recommends | ① | ② | ③ | ④ | ⑤ |
| 2) | If a friend or family recommends, I would have colonoscopy | ① | ② | ③ | ④ | ⑤ |
| 3) | If mass media (TV, radio, etc.) promote colonoscopy, I would have colonoscopy | ① | ② | ③ | ④ | ⑤ |
| 4) | If I have CRC related symptoms, I would have colonoscopy | ① | ② | ③ | ④ | ⑤ |
| 5) | I'm worried about my health, so I would have colonoscopy | ① | ② | ③ | ④ | ⑤ |
| 6) | If my family or neighbors have CRC, I would have colonoscopy | ① | ② | ③ | ④ | ⑤ |

Q6. The questions below are about your future chance of having colonoscopy. Please **tick √ the most appropriate response.**

| **Questions** | | **Strongly disagree** | **Disagree** | **Neutral** | **Agree** | **Strongly agree** |
| --- | --- | --- | --- | --- | --- | --- |
| 1) | If colonoscopy is made available as the primary CRC screening modality under the NCSP, would you like to undergo colonoscopy? | ① | ② | ③ | ④ | ⑤ |

| **Section 2. General information** |
| --- |

| **※**  Please **tick √ the most appropriate response.** |
| --- |

Q7-1. What is your age? ____________

Q7-2. What is the highest degree or level of education that you have completed?

1. Primary school ② Secondary school ③ High school degree or equivalent

④ Bachelor’s degree or Master’s degree ⑤ Others ( )

Q7-3. What is your marital status?

① Married ② Not married ③ Widowed/Divorced/Separated ④ Others ( )

Q7-4. Are you employed?

① Yes ② No

Q7-5. What is your monthly household income?

1. Less than $99 ② $1,000~$1,499 ③ $1,500~$1,999

④ $2,000~$2,499 ⑤ $2,500~$2,999 ⑥ $3,000~$3,499

⑦ $3,500~$3,999 ⑧ $4,000~$4,499 ⑨ $4,500~$4,999

⑩ $5,000~$6,999 ⑪ $7,000~$9,999 ⑫ Over $10,000

⑬ None

Q7-6. Are you currently holding a private insurance? (cancer insurance and etc.)

① Yes ② No ③ Don’t know

Q7-7. What is your general interest in health?

1. Highly interested ② Neutral ③ Not interested

Q7-8. Do you exercise regularly?

1. Regularly
2. Sometimes
3. Not at all

Q7-9. What is your current smoking status?

1. Current smoker
2. Ex-smoker
3. Never smoker

Q7-10. Have you been diagnosed with any following diseases? (You may select multiple answers)

1. Hypertension ② Diabetes ③ Chronic kidney disease

④ Cerebrovascular diseases (e.g. stroke) ⑤ Inflammatory bowel disease

⑥ None ⑦ Others ( )

| **Section 3. History of cancer and CRC screening** |
| --- |

| **※ Please tick √ the most appropriate response** |
| --- |

Q8. Have you been diagnosed with cancer?

① Yes (☞ Move to Q8-1) ② No (☞ Move to Q9)

Q8-1. If you have been diagnosed with cancer, what is the type of cancer?

|  |
| --- |

Q9. Have any of your family members or relatives been diagnosed with cancer or died of cancer?

① Yes (☞ Move to Q9-1) ② No (Move to ☞ Q10)

Q9-1. Who is your family members or relatives who have been diagnosed with cancer or died of cancer? (You may select multiple answers)

① Father ② Mother ③ Grand parents ④ Brothers/Sisters ⑤ Others ( )

Q9-2. What is the cancer type that your family members or relatives who have been diagnosed with or died of ? (If you selected multiple answers in Q 9-1, Please answer by per person)

|  |
| --- |

Q10. Are there any medical staff, family, or friends who recommended colonoscopy screening?

1. Yes (☞ Move to Q10-1) ② No (☞ Move to Q11)

Q10-1. Who recommended colonoscopy screening? (You may select multiple answers)

1. Medical staff ② Family ③ Friends ④ Others ( )

Q11. Have you ever undergone colonoscopy for colorectal cancer screening?

1. Yes ② No (☞ End of survey)

Q11-1. Have you undergone screening colonoscopy during last 10 years?

1. Yes ② No (☞ End of survey)

Q11-2. If you have undergone colonoscopy during last 10 years, how regularly do you have colonoscopy?

① Regularly (every 5 to10 years)

② Not regularly, whenever I remember

③ Not regularly
