# Supplementary material for: Intentions to undergo primary screening with colonoscopy under the National Cancer Screening Program in Korea
Source: PLoS One. 2021 Feb 24;16(2):e0247252. doi: 10.1371/journal.pone.0247252 (PMC7904222; doi:10.1371/journal.pone.0247252)
Supplement: S2 File — (DOCX) [file pone.0247252.s004.docx]

**S2 File.**

| **Section1. 대장암과 대장암 검진 방법에 대한 건강신념 측정항목** |
| --- |

1. 대장암에 관한 사항

문1. 대장암에 대한 인지된 민감성에 관한 질문입니다. 아래의 문장을 읽고 귀하의 생각과 일치하는 곳에 √ 표시해 주십시오.

| **문항내용** | | **전혀 그렇지 않다** | **그렇지 않다** | **보통 이다** | **그렇다** | **매우 그렇다** |
| --- | --- | --- | --- | --- | --- | --- |
| 1) | 나는 대장암에 걸릴 수 있다고 생각한다 | ① | ② | ③ | ④ | ⑤ |
| 2) | 나는 10년 이내에 대장암에 걸릴 가능성이 있다 | ① | ② | ③ | ④ | ⑤ |
| 3) | 나는 대장암에 걸릴 위험요인이 많다 | ① | ② | ③ | ④ | ⑤ |
| 4) | 나는 다른 사람에 비해 대장암에 걸릴 가능성이 크다고 생각한다 | ① | ② | ③ | ④ | ⑤ |

문2. 다음은 대장암에 대한 인지된 **심각성**에 관한 질문입니다.
아래의 문장을 읽고 **귀하의 생각과 일치하는 곳에 √ 표시해 주십시오.**

| **문항내용** | | **전혀 그렇지 않다** | **그렇지 않다** | **보통 이다** | **그렇다** | **매우 그렇다** |
| --- | --- | --- | --- | --- | --- | --- |
| 1) | 대장암에 걸릴지도 모른다는 생각이 나를 두렵게 한다 | ① | ② | ③ | ④ | ⑤ |
| 2) | 대장암에 걸리면 이로 인해 경험할 문제들이 오랫동안 지속될 것이다 | ① | ② | ③ | ④ | ⑤ |
| 3) | 대장암에 걸리면 가족과 대인관계에 악영향을 미칠 것이다 | ① | ② | ③ | ④ | ⑤ |
| 4) | 대장암에 걸린다면 나의 인생 전체가 변할 것 같다 | ① | ② | ③ | ④ | ⑤ |
| 5) | 만약 대장암이 발병하여 진행된다면, 나는 5년 이내에 죽을 수 있다고 생각한다 | ① | ② | ③ | ④ | ⑤ |
| 6) | 대장암은 조기에 발견되더라도 심각한 질병이다 | ① | ② | ③ | ④ | ⑤ |
| 7) | 대장암을 치료하는 것은 많은 비용이 든다 | ① | ② | ③ | ④ | ⑤ |

**B. 대장내시경에 관한 사항**

| 【참고】 대장내시경 검사란? |  |  |
| --- | --- | --- |
|  |  |  |
| **항문에 카메라가 달린 내시경관을 넣어서 대장을 직접 관찰하는 검사입니다.** 대장내시경은 **정확도가 높고, 의심스러운 병변이 발견된 경우 바로 조직검사가 가능하며, 작은 용종의 경우 바로 내시경적 제거가 가능**합니다. 하지만 대장내시경은 검사를 위해서 장을 깨끗이 비워야 하기 때문에 **장정결 과정이 힘들고**, 검사 중에는 대장에 가스를 주입하고 대장부위 끝까지 내시경관이 들어가므로 **검사과정에 통증이 유발되지만, 수면내시경을 시행해서 이런 통증을 감소시킬 수 있습니다.**  검사 부작용으로 장천공(10,000명당 3.8명)이 발생할 수 있고, 조직검사를 시행하는 경우 출혈이 생길 수 있으며, 장에 가스가 차서 복통을 유발할 수 있습니다. 고령의 환자나 고혈압 등이 있는 고위험 군에서는 검사과정에서 급성 심혈관질환(급성 심근경색)이 발생할 수도 있습니다. | | |

문3. 다음은 대장암에 대한 인지된 **유익성**에 관한 질문입니다.
아래의 문장을 읽고 **귀하의 생각과 일치하는 곳에 √ 표시해 주십시오.**

| **문항내용** | | **전혀 그렇지 않다** | **그렇지 않다** | **보통 이다** | **그렇다** | **매우 그렇다** |
| --- | --- | --- | --- | --- | --- | --- |
| 1) | 대장암을 조기에 발견한다면, 나는 살 수 있을 것이다 | ① | ② | ③ | ④ | ⑤ |
| 2) | 대장내시경 검사는 대장암의 조기발견에 도움을 줄 것이다 | ① | ② | ③ | ④ | ⑤ |
| 3) | 조기에 발견한다면, 대장암 치료는 어렵지 않을 수도 있다 | ① | ② | ③ | ④ | ⑤ |
| 4) | 대장내시경 검사는 대장암에 대한 걱정을 줄여줄 것이다 | ① | ② | ③ | ④ | ⑤ |
| 5) | 대장내시경 검사는 대장암으로 사망할 가능성을 감소시킬 것이다 | ① | ② | ③ | ④ | ⑤ |

문4. 다음은 대장암에 대한 인지된 **장애성**에 관한 질문입니다.
아래의 문장을 읽고 **귀하의 생각과 일치하는 곳에 √ 표시해 주십시오.**

| **문항내용** | | **전혀 그렇지 않다** | **그렇지 않다** | **보통 이다** | **그렇다** | **매우 그렇다** |
| --- | --- | --- | --- | --- | --- | --- |
| 1) | 이상소견이 발견될까 봐 대장내시경 검사를 받는 것이 두렵다 | ① | ② | ③ | ④ | ⑤ |
| 2) | 대장내시경 검사를 받는 것은 부끄럽고 민망하다 | ① | ② | ③ | ④ | ⑤ |
| 3) | 대장내시경 검사를 받을 시간이 없다 | ① | ② | ③ | ④ | ⑤ |
| 4) | 검사비용 때문에 대장내시경 검사를 받을 수가 없다 | ① | ② | ③ | ④ | ⑤ |
| 5) | 나는 아무 이상이 없기 때문에 대장내시경을 받을 필요가 없다 | ① | ② | ③ | ④ | ⑤ |
| 6) | 대장내시경 검사가 어떤 것인지 잘 모르기 때문에 검사를 받는 것이 두렵다 | ① | ② | ③ | ④ | ⑤ |
| 7) | 대장내시경 검사는 아프다 | ① | ② | ③ | ④ | ⑤ |
| 8) | 대장내시경 검사 전 식이조절과 장 세척 (하제복용/관장)하는 것이 힘들어서 검사를 받을 수가 없다 | ① | ② | ③ | ④ | ⑤ |
| 9) | 장출혈이나 장손상 같은 부작용이 생길까 봐 대장내시경 검사를 받는 것이 두렵다 | ① | ② | ③ | ④ | ⑤ |
| 10) | 검사를 위해 오고 가는 차편에 문제가 있어 대장내시경 검사를 받을 수가 없다 | ① | ② | ③ | ④ | ⑤ |
| 11) | 대장내시경 검사를 믿을 수 없다 | ① | ② | ③ | ④ | ⑤ |

문5. 다음은 대장암 **검진계기**에 관한 질문입니다.
아래의 문장을 읽고 **귀하의 생각과 일치하는 곳에 √ 표시해 주십시오.**

| **문항내용** | | **전혀 그렇지 않다** | **그렇지 않다** | **보통 이다** | **그렇다** | **매우 그렇다** |
| --- | --- | --- | --- | --- | --- | --- |
| 1) | 만약 의사가 권고한다면 대장내시경 검진을 받을 의향이 있다 | ① | ② | ③ | ④ | ⑤ |
| 2) | 만약 친구나 가족이 추천한다면 대장내시경 검진을 받을 의향이 있다 | ① | ② | ③ | ④ | ⑤ |
| 3) | 만약 대중매체 (TV, 라디오 등)에서 대장내시경에 대해 홍보한다면 대장내시경 검사를 받을 의향이 있다 | ① | ② | ③ | ④ | ⑤ |
| 4) | 대장암 관련 이상증세를 느낀다면, 대장내시경 검사를 받을 것이다 | ① | ② | ③ | ④ | ⑤ |
| 5) | 나의 건강상태가 염려되기 때문에 대장내시경 검사를 받겠다 | ① | ② | ③ | ④ | ⑤ |
| 6) | 만약 가족이나 주변인 중 대장암을 앓고 있는 사람이 있다면, 나는 대장내시경 검사를 받으려고 할 것이다 | ① | ② | ③ | ④ | ⑤ |

문6. 향후 대장암 **검진 가능성**에 관한 질문입니다.
아래의 문장을 읽고 **귀하의 생각과 일치하는 곳에 √ 표시해 주십시오.**

| **문항내용** | | **전혀 그렇지 않다** | **그렇지 않다** | **보통 이다** | **그렇다** | **매우 그렇다** |
| --- | --- | --- | --- | --- | --- | --- |
| 1) | 만약 국가 암 검진으로 대장내시경을 제공한다면, 대장내시경으로 검진을 받겠다 | ① | ② | ③ | ④ | ⑤ |

| **Section2. 응답자 일반사항** |
| --- |

| **【설명】 일반사항** |  |  |
| --- | --- | --- |
|  |  |  |
| **※ 다음은 귀하의 일반적인 사항에 관한 질문입니다. 해당하는 항목에 직접 기록하거나 √ 표시 해 주십시오.** | | |

문7-1. 귀하의 **최종학력**은 무엇입니까?

1. 초등학교 졸업 ② 중학교 졸업 ③ 고등학교 졸업

④ 대학교 또는 대학원 졸업 ⑤ 기타( )

문7-2. 귀하의 **결혼 상태**는 무엇입니까?

① 미혼 ② 기혼 ③ 사별/이혼/별거 ④ 기타( )

문7-3. 귀하는 **직업**이 있습니까?

① 있다 ② 없다

문7-4. 귀 **가정의 한 달 수입**은 어느 정도 입니까?

① 99만원 이하 ② 100~149만원 ③ 150~199만원

④ 200~249만원 ⑤ 250~299만원 ⑥ 300~349만원

⑦ 350~399만원 ⑧ 400~449만원 ⑨ 450~499만원

⑩ 500~699만원 ⑪ 700~999만원 ⑫ 1,000만원 이상

⑬ 소득 없음

문7-5. 귀하는 현재 의료비를 보조해 주는 **민영보험(암보험, 실비보험 등) 상품**에 가입되어 있습니까?

① 그렇다 ② 아니다 ③ 모름

문7-6. 귀하는 평소 **건강에 대한 관심** 정도가 어떠하십니까?

① 관심이 아주 많다 ② 보통이다 ③ 관심이 거의 없다

문7-7. 귀하는 **평소 규칙적인 운동**을 하고 계십니까?

① 규칙적인 운동을 하고 있다 ② 가끔 생각날 때마다 하고 있다 ③ 운동을 하지 않는다

문7-8. 귀하의 현재 흡연 상태는 어떻습니까?

① 현재 담배를 피우고 있다

② 과거에는 피웠으나 현재는 피우지 않는다

③ 담배를 피운 적이 없다

문7-9. 다음 중에 이전에 **진단 받았던 질환**이 있습니까? (복수 선택 가능)

① 고혈압 ② 당뇨 ③ 만성콩팥병 ④ 뇌혈관 질환(뇌졸중 등)

1. 염증성 장 질환 ⑥ 없음 ⑦ 기타( )

| **Section4. 암 병력과 대장암 검진관련 항목** |
| --- |

| **※ 다음은 귀하의 암 병력과 대장암 검진에 관한 질문입니다 해당하는 항목에 직접 기록하거나 √ 표시 해 주십시오.** |
| --- |

문8. 귀하는 암 진단을 받은 적이 있으십니까?

① 있다(☞문8-1로 이동) ② 없다(☞문9로 이동)

문8-1. 진단 받은 암 유형은 무엇입니까?

|  |
| --- |

문9. 가족, 친척 중에 암 진단을 받거나 암으로 사망하신 분이 있으십니까?

1. 있다(☞문9-1로 이동) ② 없다(☞문10으로 이동)

문9-1. 가족, 친척 중에 암 진단을 받거나 암으로 사망하신 분과 본인과의 관계는 무엇입니까? (중복응답 가능)

① 부 ② 모 ③ 조부모 ④ 형제/자매 ⑤ 기타( )

문9-2. 가족, 친척 중에 암 진단을 받거나 암으로 사망하신 분이 진단받은 암 유형은 무엇입니까?

(문 9-1번 중복 응답 시 본인과의 관계별로 정리하여 작성)

|  |
| --- |

문10. 의료인, 가족, 친구 등 주위에서 대장내시경 검진을 권하는 사람이 있습니까?

① 있다(☞문10-1로 이동) ② 없다(☞문11로 이동)

문10-1. (문10에서) 대장내시경 검진을 권하는 사람은 누구입니까?(중복응답 가능)

① 의료인 ② 가족 ③ 친구 ④ 기타( )

문11. 귀하는 평생 동안 대장암 검진을 위해 대장내시경 검사를 한 번이라도 받아 본 적이 있습니까?

① 받아본 적이 있다 ② 받아본 적이 없다(☞설문종료)

문11-1. 최근 10년 이내에 대장암 검진을 위해 대장내시경 검사를 받은 적이 있습니까?

① 있다 ② 없다(☞설문종료)

문11-2. 최근 10년 이내 받아본 적이 있다면, 대장내시경 검사의 실시주기는 어떻게 되십니까?

① 정기적(5~10년 단위)으로 검사를 받고 있다

② 정기적으로는 아니지만 생각날 때마다 검사를 받고 있다

③ 정기적으로 받고 있지 않다
