# Supplementary material for: Intentions to undergo primary screening with colonoscopy under the National Cancer Screening Program in Korea
Source: PLoS One. 2021 Feb 24;16(2):e0247252. doi: 10.1371/journal.pone.0247252 (PMC7904222; doi:10.1371/journal.pone.0247252)
Supplement: S1 Table — (DOCX) [file pone.0247252.s005.docx]

S1 Table.

| **Items** | **Factor 1** | **Factor 2** | **Factor 3** | **Factor 4** | **Factor 5** |
| --- | --- | --- | --- | --- | --- |
| **Factor 1 (Perceived susceptibility)** |  |  |  |  |  |
| It is likely that I will get CRC. | **0.73** | 0.33 | 0.15 | -0.01 | 0.08 |
| It is likely that I will get CRC within the next 10 years. | **0.82** | 0.15 | 0.03 | 0.09 | 0.06 |
| I have many risk factors for CRC. | **0.84** | 0.21 | 0.02 | 0.07 | 0.08 |
| I have a higher probability of developing CRC than others. | **0.85** | 0.15 | -0.01 | 0.12 | 0.11 |
| **Factor 2 (Perceived susceptibility)** |  |  |  |  |  |
| The thought of getting CRC scares me. | **0.60** | 0.36 | 0.04 | 0.17 | 0.23 |
| Problems I would experience with CRC would last a long time. | 0.32 | **0.71** | 0.16 | 0.06 | 0.13 |
| CRC will negatively affect family and social relationships. | 0.23 | **0.78** | 0.14 | 0.05 | 0.08 |
| If I had CRC, my whole life would change. | 0.17 | **0.82** | 0.16 | 0.01 | 0.09 |
| If I had CRC, I would not live longer than 5 years. | 0.31 | **0.66** | -0.06 | 0.15 | 0.13 |
| CRC is deadly even if it is found early. | 0.27 | **0.61** | -0.18 | 0.21 | 0.13 |
| CRC treatment is expensive. | 0.17 | **0.74** | 0.05 | 0.11 | 0.07 |
| **Factor 3 (Perceived benefits)** |  |  |  |  |  |
| Finding CRC early will increase my chance of survival. | -0.01 | 0.06 | **0.76** | -0.12 | 0.15 |
| Colonoscopy will help me to find CRC early. | 0.02 | 0.27 | **0.70** | -0.17 | 0.21 |
| Treatment for CRC is not difficult if it is found early. | 0.05 | -0.09 | **0.79** | -0.01 | 0.14 |
| Colonoscopy will reduce concerns about CRC. | 0.10 | 0.06 | **0.78** | -0.10 | 0.20 |
| Colonoscopy will decrease my chances of dying from CRC. | 0.04 | 0.11 | **0.79** | -0.12 | 0.19 |
| **Factor 4 (Perceived barriers)** |  |  |  |  |  |
| I am afraid I will find out there is something wrong with me. | 0.23 | 0.25 | -0.05 | **0.58** | -0.07 |
| Colonoscopy is embarrassing. | 0.04 | 0.19 | -0.03 | **0.68** | -0.14 |
| I do not have enough time to have a colonoscopy. | 0.20 | -0.04 | -0.10 | **0.68** | -0.06 |
| I can’t afford a colonoscopy. | 0.09 | 0.04 | -0.18 | **0.70** | 0.02 |
| I do not need a colonoscopy because nothing is wrong with me. | -0.01 | -0.12 | -0.12 | **0.67** | -0.19 |
| I'm afraid to have a colonoscopy because I do not understand what will be done in the test. | 0.04 | 0.04 | -0.13 | **0.76** | -0.08 |
| Colonoscopy is painful. | 0.02 | 0.19 | 0.02 | **0.71** | -0.19 |
| Preparing for a colonoscopy (bowel preparation/diet restriction) is too difficult. | 0.06 | 0.25 | 0.08 | **0.65** | -0.21 |
| I'm afraid of getting a colonoscopy because of possible complications, such as intestinal bleeding or intestinal damage. | 0.04 | 0.26 | 0.02 | **0.73** | -0.09 |
| It is difficult to get transportation to have a colonoscopy. | 0.10 | -0.13 | -0.24 | **0.74** | 0.05 |
| I do not trust colonoscopy. | 0.14 | -0.10 | -0.28 | **0.67** | -0.07 |

**S1 Table (Continue)**

| **Items** | **Factor 1** | **Factor 2** | **Factor 3** | **Factor 4** | **Factor 5** |
| --- | --- | --- | --- | --- | --- |
| **Factor 5 (Cues to action)** |  |  |  |  |  |
| I would have a colonoscopy if a doctor recommends it. | 0.06 | 0.24 | 0.38 | -0.24 | **0.60** |
| If a friend or family recommends it, I would have a colonoscopy. | 0.11 | 0.10 | 0.21 | -0.14 | **0.79** |
| If mass media (TV, radio, etc.) promotes colonoscopy, I would have it. | 0.15 | -0.02 | 0.12 | -0.03 | **0.81** |
| If I have CRC-related symptoms, I would have a colonoscopy. | -0.07 | 0.35 | 0.43 | -0.24 | **0.46** |
| I'm worried about my health, so I would have a colonoscopy. | 0.17 | 0.11 | 0.19 | -0.12 | **0.73** |
| If my family or neighbors have CRC, I would have a colonoscopy. | 0.04 | 0.27 | 0.27 | -0.10 | **0.66** |
