# Supplementary material for: Intentions to undergo primary screening with colonoscopy under the National Cancer Screening Program in Korea
Source: PLoS One. 2021 Feb 24;16(2):e0247252. doi: 10.1371/journal.pone.0247252 (PMC7904222; doi:10.1371/journal.pone.0247252)
Supplement: S2 Table — SD, standard deviations. (DOCX) [file pone.0247252.s006.docx]

S2 Table.

| **Factors** | **No. of items** | **Mean**$\boldsymbol{\pm}$**SD** | **Min** | **Max** | **Cronbach’s**  **alpha** |
| --- | --- | --- | --- | --- | --- |
| Perceived susceptibility | 4 | 3.05$\pm$0.76 | 1 | 5 | 0.88 |
| Perceived severity | 4 | 3.74$\pm$0.77 | 1 | 5 | 0.86 |
| Perceived benefits | 5 | 4.01$\pm$0.57 | 1 | 5 | 0.87 |
| Perceived barriers | 5 | 2.76$\pm$0.78 | 1 | 5 | 0.83 |
| Cues to action | 3 | 3.59$\pm$0.68 | 1 | 5 | 0.80 |

SD, Standard Deviations.
