# Supplementary material for: Intentions to undergo primary screening with colonoscopy under the National Cancer Screening Program in Korea
Source: PLoS One. 2021 Feb 24;16(2):e0247252. doi: 10.1371/journal.pone.0247252 (PMC7904222; doi:10.1371/journal.pone.0247252)
Supplement: S3 Table — (DOCX) [file pone.0247252.s007.docx]

S3 Table.

| **Variable** | **Total** | **No colonoscopy screening within 10 years** | **Colonoscopy screening within 10 years** | ***P* value**^a)^ |
| --- | --- | --- | --- | --- |
| **Total** | 800 (100) | 423 (52.9) | 377 (47.1) |  |
| **Age group (years)** |  |  |  |  |
| Mean$\pm SD$ | 58.0$\pm7.2$ | 57.0$\pm6.8$ | 59.1$\pm7.4$ | <0.001 |
| 45-54 | 319 (39.9) | 197 (46.6) | 122 (32.4) | <0.001 |
| 55-64 | 312 (39.0) | 155 (36.6) | 157 (41.6) |  |
| 65-78 | 169 (21.1) | 71 (16.8) | 98 (26.0) |  |
| **Residential area** |  |  |  |  |
| Metropolitan | 373 (46.6) | 198 (46.8) | 175 (46.4) | 0.912 |
| Non-metropolitan | 427 (53.4) | 225 (53.2) | 202 (53.6) |  |
| **Sex** |  |  |  |  |
| Male | 393 (49.1) | 187 (44.2) | 206 (54.6) | 0.003 |
| Female | 407 (50.9) | 236 (55.8) | 171 (45.4) |  |
| **Years of education** |  |  |  |  |
| 6-12 years | 243 (30.4) | 146 (34.5) | 97 (25.7) | 0.007 |
| More than 13 years | 557 (69.6) | 277 (65.5) | 280 (74.3) |  |
| **Monthly household income** |  |  |  |  |
| Less than $2,999 | 202 (25.3) | 135 (31.9) | 67 (17.8) | <0.001 |
| $3,000~$4,999 | 294 (36.8) | 156 (36.9) | 138 (36.6) |  |
| More than $5,000 | 304 (38.0) | 132 (31.2) | 172 (45.6) |  |
| **Employment status** |  |  |  |  |
| Unemployed | 193 (24.1) | 119 (28.1) | 74 (19.6) | 0.005 |
| Employed | 607 (75.9) | 304 (71.9) | 303 (80.4) |  |
| **Physical activity** |  |  |  |  |
| None | 139 (17.4) | 83 (19.6) | 56 (14.9) | 0.042 |
| Moderate | 416 (52.0) | 225 (53.2) | 191 (50.7) |  |
| Regular | 245 (30.6) | 115 (27.2) | 130 (34.4) |  |
| **Private cancer insurance** |  |  |  |  |
| No | 185 (23.1) | 111 (26.2) | 74 (19.6) | 0.027 |
| Yes | 615 (76.9) | 312 (73.8) | 303 (80.4) |  |
| **Current smoking status** |  |  |  |  |
| No | 577 (72.1) | 318 (75.2) | 259 (68.7) | 0.041 |
| Yes | 223 (27.9) | 105 (24.8) | 118 (31.3) |  |
| **Chronic disease** |  |  |  |  |
| No | 473 (59.1) | 273 (64.5) | 200 (53.1) | 0.001 |
| Yes | 327 (40.9) | 150 (35.5) | 177 (47.0) |  |

Chronic disease, being diagnosed with any of following diseases: hypertension, diabetes, chronic kidney disease, cerebrovascular disease, and inflammatory bowel disease.

^a)^Comparison of frequencies between not up-to-date colonoscopy and up-to-date colonoscopy groups using the chi-squire test.
