# Supplementary material for: Intentions to undergo primary screening with colonoscopy under the National Cancer Screening Program in Korea
Source: PLoS One. 2021 Feb 24;16(2):e0247252. doi: 10.1371/journal.pone.0247252 (PMC7904222; doi:10.1371/journal.pone.0247252)
Supplement: S4 Table — aOR, adjusted odds ratio; 95% CI, 95% confidence interval; Chronic disease, being diagnosed with any of the following diseases: hypertension, diabetes, chronic kidney disease, cerebrovascular disease, and inflammatory bowel disease. * OR per one score increase. (DOCX) [file pone.0247252.s008.docx]

S4 Table.

| **Variable** | **No colonoscopy screening within 10 years** | | **Colonoscopy screening within 10 years** | |
| --- | --- | --- | --- | --- |
|  | **aOR** | **95% CI** | **aOR** | **95% CI** |
| **Age group (years)** |  |  |  |  |
| 45-54 | 1.0 | reference | 1.0 | reference |
| 55-64 | 0.89 | 0.51-1.57 | 1.13 | 0.42-3.00 |
| 65-78 | 0.61 | 0.29-1.26 | 1.85 | 0.54-6.31 |
| **Residential area** |  |  |  |  |
| Metropolitan | 1.0 | reference | 1.0 | reference |
| Non-metropolitan | 1.26 | 0.76-2.09 | 0.93 | 0.40-2.16 |
| **Sex** |  |  |  |  |
| Male | 1.0 | reference | 1.0 | reference |
| Female | 0.96 | 0.53-1.76 | 0.65 | 0.26-1.59 |
| **Years of education** |  |  |  |  |
| 6-12 years | 1.0 | reference | 1.0 | reference |
| More than 13 years | 0.83 | 0.47-1.47 | 1.06 | 0.41-2.74 |
| **Monthly household income** |  |  |  |  |
| Less than $2,999 | 1.0 | reference | 1.0 | reference |
| $3,000~$4,999 | 1.12 | 0.60-2.10 | 2.11 | 0.72-6.19 |
| More than $5,000 | 0.85 | 0.43-1.70 | 2.33 | 0.76-7.12 |
| **Employment status** |  |  |  |  |
| Unemployed | 1.0 | reference | 1.0 | reference |
| Employed | 1.19 | 0.64-2.19 | 1.07 | 0.38-3.01 |
| **Physical activity** |  |  |  |  |
| None at all | 1.0 | reference | 1.0 | reference |
| Moderate | 1.17 | 0.60-2.27 | 1.28 | 0.39-4.20 |
| Regular | 1.47 | 0.68-3.17 | 0.99 | 0.27-3.60 |
| **Private cancer insurance** |  |  |  |  |
| No | 1.0 | reference | 1.0 | reference |
| Yes | 1.36 | 0.76-2.41 | 1.07 | 0.37-3.11 |
| **Smoking status** |  |  |  |  |
| No | 1.0 | reference | 1.0 | reference |
| Yes | 0.93 | 0.48-1.80 | 1.21 | 0.47-3.10 |
| **Chronic Disease** |  |  |  |  |
| No | 1.0 | reference | 1.0 | reference |
| Yes | 1.12 | 0.64-1.95 | 1.30 | 0.57-2.97 |
| **Perceived susceptibility^a)^** | 0.93 | 0.63-1.40 | 0.54 | 0.24-1.22 |
| **Perceived severity ^a)^** | 1.66 | 1.11-2.49 | 1.42 | 0.71-2.81 |
| **Perceived benefit ^a)^** | 2.25 | 1.31-3.86 | 5.28 | 2.20-12.65 |
| **Perceived barrier ^a)^** | 0.67 | 0.43-1.07 | 0.73 | 0.37-1.44 |
| **Cues to action ^a)^** | 8.48 | 4.77-15.09 | 8.00 | 3.33-19.22 |

aOR, adjusted Odds Ratio; 95% CI, 95% Confidence Interval; Chronic disease, being diagnosed with any of following diseases: hypertension, diabetes, chronic kidney disease, cerebrovascular disease, and inflammatory bowel disease. ^a)^OR per one score increase.
